# Is grip strength a marker for cognitive function, neurodegeneration, and resilience?

**DOI:** 10.1007/s11357-025-02057-y

**Published:** 2025-12-23

**Authors:** Alexander Ivan B. Posis, Hilary L. Colbeth, Mihika S. Deshpande, Kristen M. George, Batool Rizvi, Rifat B. Alam, Ruijia Chen, Dan M. Mungas, Paola Gilsanz, María M. Corrada, Rachel A. Whitmer

**Affiliations:** 1 Department of Public Health Sciences, University of California, Davis, Medical Sciences 1-C, One Shield’s Ave, Davis, CA 95616 USA; 2 Department of Epidemiology, Boston University School of Public Health, Boston, MA USA; 3 Department of Neurology, University of California, Davis, Davis, CA USA; 4 Kaiser Permanente Northern California Division of Research, Pleasanton, CA USA; 5 Department of Neurology, University of California, Irvine, Irvine, CA USA; 6 Department of Epidemiology and Biostatistics, University of California, Irvine, Irvine, CA USA

**Keywords:** Cognitive decline, Neuroimaging, Physical function, Physical performance

## Abstract

Assessing whether grip strength is associated with cognitive decline and brain health could help identify a target to prevent or delay cognitive impairment. Therefore, we tested whether grip strength is associated with brain health as indicated by magnetic resonance imaging and domain specific cognitive decline. We also assessed whether grip strength was a cognitive resilience proxy using moderation tests. This study included cognitive and neuroimaging data from 861 participants in three harmonized cohorts. We fit multivariable linear mixed-effects and linear regression models to test associations between grip strength, neuroimaging markers, and cognition. We also tested moderation by gender and age. Greater grip strength was associated with slower verbal episodic memory decline and greater total gray matter volume. Associations of grip strength with executive function, total hippocampal volume, and white matter hyperintensities were not significant, but in the expected direction. Age moderated associations of grip strength and white matter hyperintensities. Grip strength was a significant proxy for cognitive resilience related to white matter hyperintensity volume and verbal episodic memory decline only. Future research should examine other measures of physical performance that are associated with later-life cognitive and brain health.

**Supplementary Information:**

The online version contains supplementary material available at https://doi.org/10.1007/s11357-025-02057-y.

## Introduction

Greater physical performance is associated with lower risk of dementia and slower rate of cognitive decline [1–4]. Physical performance can be measured by hand grip strength. Increased grip strength is associated with greater brain integrity and cognitive outcomes [5–14]. However, this evidence is primarily based on non-Hispanic White study populations of narrow age ranges, limiting generalizability.

Cardiometabolic and vascular risk factors are associated with markers of poor brain health, such as lower total gray matter and hippocampal volume, as well as the presence of white matter hyperintensities (WMHs) [15–17]. These markers of poor brain health are associated with elevated risk of cognitive decline and dementia [18–20]. Physical activity and performance levels, among other lifestyle factors, are also implicated in cardiovascular, inflammatory, and metabolic processes [21]. For instance, weaker grip strength is associated with greater risk of cardiovascular disease and cardiovascular disease–related mortality [22, 23]. This potentially links grip strength to brain and cognitive health. However, connecting grip strength with brain and cognitive outcomes requires further elucidation across multiple settings and under certain identifiability conditions to infer causality [24].

**Fig. 1:**
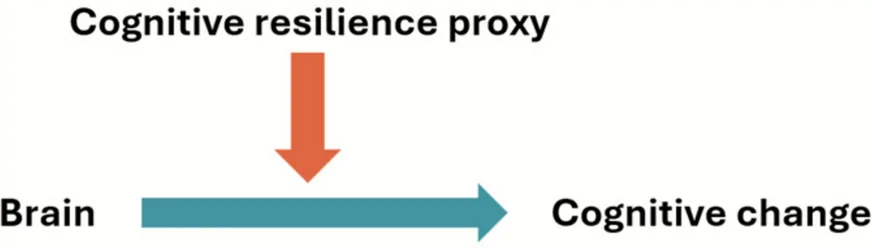
Framework for testing a theorized proxy of cognitive resilience using moderation. Theorized proxies of cognitive resilience should moderate associations between brain health and cognitive decline

There is growing interest regarding why some individuals are more resilient to neuropathology-related cognitive decline compared with others [25]. Individuals are considered to demonstrate resilience if they have better than expected cognitive function given the presence of brain pathology that would suggest lower levels of cognitive function [25, 26]. Proxies for cognitive resilience can be identified using tests of moderation [25–27]. Meaning, if the association between brain health with cognitive change differs across a level of a covariate, it may be theorized as a cognitive resilience proxy (see Fig. 1). Grip strength may be a proxy for cognitive resilience given its association with brain and cognitive outcomes [5–14]. Testing grip strength as a moderator using this resilience framework [25] may provide further insight into later life brain and cognitive health.

Grip strength is easily measured and potentially modifiable, even among older adults [28, 29], thus is an important marker to monitor in relation to cognitive impairment and neurodegeneration. The objective of this prospective cohort study is to test whether grip strength is associated with brain health as indicated by magnetic resonance imaging (MRI) and domain specific cognitive decline. We hypothesized that greater grip strength is associated with better brain health outcomes and slower cognitive decline. We also tested differences by age and gender [6]. Lastly, we assessed whether grip strength was a proxy for cognitive resilience by testing grip strength-by-brain interactions in relation to cognitive decline.

## Methods

### Study population

This prospective cohort study harmonized data from three ongoing cohort studies of long-term Kaiser Permanente Northern California (KPNC) members residing in the San Francisco Bay and Sacramento areas: (1) the Kaiser Healthy Aging and Diverse Life Experiences (KHANDLE) study, (2) the Study of Healthy Aging in African Americans (STAR), and (3) the *LifeAfter90* study. Full study details are described elsewhere [1, 30–35]. Briefly, KHANDLE includes ethnoracially diverse adults aged 65 years or older, STAR includes Black older adults aged 50 years or older, and *LifeAfter90* includes adults aged 90 years or older. Inclusion criteria at time of enrollment for all studies included the capacity to provide informed consent; no diagnosis of dementia, other neurodegenerative disease, or dialysis in their electronic medical record; and not in hospice care. For follow-up, there were up to three study visits in KHANDLE and STAR which occurred approximately every 14 months, and eight study visits for *LifeAfter90* which occurred approximately every 6 months*.* Enrolled individuals were asked if they were interested in participating in the structural magnetic resonance imaging (MRI) component of the study. Of those who were, a random sample of 25% of participants within each study were selected for MRI. Protocols for all three studies were reviewed and approved by the KPNC and University of California, Davis, institutional review boards. All participants provided informed consent. Research was performed in accordance with the Declaration of Helsinki.

Of the 3957 study participants across all three studies, 915 had imaging data and were considered for the present study. We then excluded 6 participants without WMH data, 41 participants missing demographic data, and 7 participants missing grip strength data, yielding a final analytic sample of 861 participants. Participants provided repeat grip strength assessments and cognitive assessments during study visits scheduled in regular intervals while their first set of brain image could have been acquired at any point during study follow-up. Therefore, we selected participants’ grip strength data at the closest study visit to imaging, which could have occurred before or after imaging. The average difference between participants’ age at grip strength assessment and MRI was − 0.29 years (range =  − 4.60, 1.41 years); our analyses adjusted for this time difference, as described below. In short, we defined baseline as the KHANDLE, STAR, or *LifeAter90* study visit that was closest to when the participant’s MRI was acquired.

### Measures

#### Grip strength

We used grip strength measures at the study visit closest to neuroimaging, which is the study baseline. Grip strength was assessed using Jamar Hydraulic Hand Dynamometers (Crawfordsville, IN, USA) across three trials with both hands and measured in kilograms (kg). For the present study, grip strength data was not available for all participants for both dominant and non-dominant hands. Therefore, we used the average of the three trials from the participants’ dominant hand. Our primary grip strength outcome was standardized using mean and standard deviation values defined by cohort (KHANDLE; STAR; *LifeAfter90*) and gender (women; men; see Supplementary Table 1).

#### MRI acquisition and processing

MRI acquisition and processing were performed using the same standardized protocol in all three cohorts. Briefly, MRIs were acquired using 3 T Siemens TrioTrim or Prisma Fit models. There were three sequences: (1) T1-weighted volumetric MP-RAGE (3DT1): repetition time (TR) = 2500 ms, echo time (TE) = 2.98 ms, inversion time (TI) = 1100 ms, 192 slices, field of view (FOV) = 256 mm, acquisition matrix = 256 × 256, slice thickness = 1 mm; (2) fluid attenuated inversion recovery (FLAIR): TR = 8800 ms, TE = 500 ms, TI = 2360 ms, 96 slices, FOV = 256 mm, acquisition matrix = 256 × 256, slice thickness = 2 mm; and (3) multi-shell DTI: TR = 6000 ms, TE = 87 ms, 48 slices, FOV = 256 mm, acquisition matrix = 96 × 96, slice thickness = 2.7 mm with a 2.7 mm gap. Diffusion weighted images were created with 13 gradient directions with gradient diffusion sensitivity of *b* = 500 s/mm^2^, 21 gradient directions with *b* = 1000 s/mm^2^, 15 gradient directions with *b* = 2000s/mm^2^, and 3 images with *b* = 0 s/mm^2^.

Total intracranial volume (ICV) segmentation and quantification were performed using an in-house convolutional neural network method, as described previously [36]. Regional gray matter volumes were calculated using non-linear co-registration of images to the Desikan-Killiany-Tourville atlas [37–39]. WMHs were segmented using a Bayesian approach estimated from FLAIR images [40]. Hippocampal masks were computed using a multi-atlas hippocampal segmentation algorithm [38].

MRI variables of interest included gray matter, hippocampal, and WMH volumes. WMH volume was log-transformed due to a non-normal distribution. All measures were corrected for ICV by regressing each region of interest volume onto ICV and calculating residual values. Residual ICV-corrected values for total gray matter, hippocampal, and WMH volumes were then Z-scored to allow for comparability.

#### Cognitive performance outcomes

The Spanish and English Neuropsychological Scales (SENAS) was used to derive indices for two cognitive domains at all study visits: (1) verbal episodic memory, and (2) executive function [41, 42]. The verbal episodic memory composite score was derived from a word list learning test [41]. The executive function composite score was derived from tasks related to category fluency, phonemic fluency, and digit-span backward and list sorting tasks for working memory [42]. The SENAS administration was modified in LifeAfter90 using shortened versions of multiple tasks [33]. For comparability across the three cohorts, verbal episodic memory and executive function scores were *z*-standardized using means and standard deviations from the baseline visit of the analytic sample.

#### Covariates

We considered sociodemographic characteristics including age at MRI, gender (men; women), race/ethnicity (African American/Black; Asian; Latine; Multiracial or other racial/ethnic group; White), highest reported education completed (≤ High School/GED; Some College or Tech/Trade School; College; Graduate School), time between grip strength and neuroimaging measurement, and cohort (KHANDLE; STAR; *LifeAfter90*) as potential confounders. Due to the COVID-19 pandemic, some assessments were conducted via phone and thus we included a covariate for interview mode (phone; in-person). To account for practice effects, we included a visit 1 indicator (yes; no) [30, 43].

### Statistical analysis

#### Population description

We described participant characteristics using median (minimum, maximum values) or mean (SD) for continuous variables or counts (percentages) for categorical variables by cohort. 

#### Associations of grip strength with cognitive decline

We fit separate multivariable linear mixed-effects models with random intercepts to test associations of grip strength with cognitive decline. All models included random intercepts and used years since baseline as the timescale. We did not include random slopes due to limited model convergence. All models adjusted for age, gender, race/ethnicity, education, interview mode, practice effects, time between grip strength and neuroimaging measurements, and cohort. For visualization, we plotted the average trajectories of cognitive decline by mean and ± 1 SD of grip strength.

To account for selection bias due to loss to follow-up, our linear mixed-effects models were fit with time-varying inverse probability of censoring weights (IPCWs) [24]. For each participant at each visit, we estimated IPCWs by taking the inverse of propensity scores for availability at each of the eight potential study visits (up to eight visits for *LifeAfter90*; up to three visits for KHANDLE and STAR), conditional on total gray matter, hippocampal and WMH volumes as well as grip strength, standardized age, gender, race/ethnicity, and education. Weights were stabilized by the probability of participation at each of the eight potential study visits, conditional on total gray matter, hippocampal and WMH volumes as well as grip strength. Time-varying IPCWs were calculated by multiplying visit-specific cumulative weights up to each visit. For all IPCW analyses, we excluded the single observation at visit 8 out of concern for weight instability.

#### Cross-sectional associations of grip strength with neuroimaging markers at baseline

We fit three separate multivariable linear regression models to test associations of grip strength with total gray matter, hippocampal, and WMH volumes. All models adjusted for age, gender, race/ethnicity, education, time between grip strength and imaging scan, and cohort. In sensitivity analyses, we repeated this analysis excluding 78 participants who had more than a ± 1 year gap between neuroimaging and grip strength measures. Compared with the 78 participants excluded due to this measurement time restriction, the 783 included in this sensitivity analysis were slightly younger (median age, 77.3 vs 78.4 years) with greater median *z*-scored grip strength (− 0.06 vs. − 0.19) as well as be more likely to be women (60.9% vs 48.7%), identify as White (21.6% vs 20.5%), and have at least a college education (49.3% vs. 44.8%; Supplementary Table 2).

#### Moderating role of gender and age

We assessed whether gender (men; women) and age (< 65 years; 65–80 years; 80–90 years; 90 + years) modified associations of grip strength with cognitive decline and imaging outcomes, using likelihood ratio tests and estimating effect modifier-stratified models. For cognitive outcomes, we compared separate multivariable linear mixed-effects models with and without a grip strength-by-modifier-by-time interaction term using likelihood ratio tests. For neuroimaging outcomes, we compared separate multivariable linear regression models with and without a grip strength-by-modifier interaction term using likelihood ratio tests. Models for testing age as a modifier did not include adjustments for race/ethnicity and cohort due to limited sample size, especially for the < 65-year-old group.

#### Testing grip strength as a proxy for cognitive resilience

Cognitive resilience proxies are posited to moderate the relationship between brain health indicators and cognitive function [25]. The use of this moderation framework is consistent with Stern et al.’s “Whitepaper: Defining and investigating cognitive reserve, brain reserve, and brain maintenance” [25]. To test whether grip strength was a cognitive resilience proxy by moderating the association of neuroimaging markers and cognitive decline, we used likelihood ratio tests to compare linear mixed-effects models with and without the grip strength-by-brain biomarker-by-time interactions. We fit separate models for each biomarker that included random intercepts and IPCW, and adjusted for age, gender, education, race/ethnicity, time between grip strength and neuroimaging measurement, practice effects, and cohort. For visualization, we plotted average trajectories of cognitive function by different levels of the neuroimaging markers, and then stratified by mean and ± 1 SD of grip strength.

All statistical analyses were conducted using R version 4.4.0 (R Core Team 2024).

## Results

### Participant characteristics

The median age of the 861 participants at the time of the scan was 77.3 years (range = 53.5, 103) (Table 1). The average follow-up time was 1.65 years (SD = 1.72; range = 0–6.57). More than half (59.8%) of participants were women. Self-reported race/ethnicity was 41.8% African American/Black, 18.5% Asian, 16.5% Latine, 1.7% Multiracial or Other racial/ethnic group, and 21.5% White. Almost half of participants had at a college or higher education (48.9%).

**Table 1:** Baseline participant characteristics by cohort

Characteristic		STAR (*N* = 225)	KHANDLE (*N* = 423)	LA90 (*N* = 213)	Overall (*N* = 861)
Age	Median [min., max.]	66.8 [53.5, 88.6]	76.1 [66.8, 89.8]	92.7 [90.5, 103]	77.3 [53.5, 103]
Gender	Men	70 (31.1%)	186 (44.0%)	90 (42.3%)	346 (40.2%)
	Women	155 (68.9%)	237 (56.0%)	123 (57.7%)	515 (59.8%)
Race/ethnicity	African American/Black	225 (100%)	87 (20.6%)	48 (22.5%)	360 (41.8%)
	Asian	-	106 (25.1%)	53 (24.9%)	159 (18.5%)
	Latine	-	102 (24.1%)	40 (18.8%)	142 (16.5%)
	Multiracial/Other	-	-	15 (7.0%)	15 (1.7%)
	White	-	128 (30.3%)	57 (26.8%)	185 (21.5%)
Education	≤ High School/GED	24 (10.7%)	54 (12.8%)	43 (20.2%)	121 (14.1%)
	Some College or Tech/Trade School	106 (47.1%)	139 (32.9%)	74 (34.7%)	319 (37.1%)
	College	50 (22.2%)	130 (30.7%)	55 (25.8%)	235 (27.3%)
	Graduate School	45 (20.0%)	100 (23.6%)	41 (19.2%)	186 (21.6%)
Grip strength^a^	Median [min., max.]	− 0.0596 [− 2.37, 2.25]	− 0.112 [− 2.77, 3.39]	− 0.139 [− 2.36, 3.20]	− 0.0644 [− 2.77, 3.39]

^a^Grip strength is *z*-scored by gender and cohort, thus resulting in positive and negative values

### Associations of grip strength with cognitive decline

**Fig. 2:**
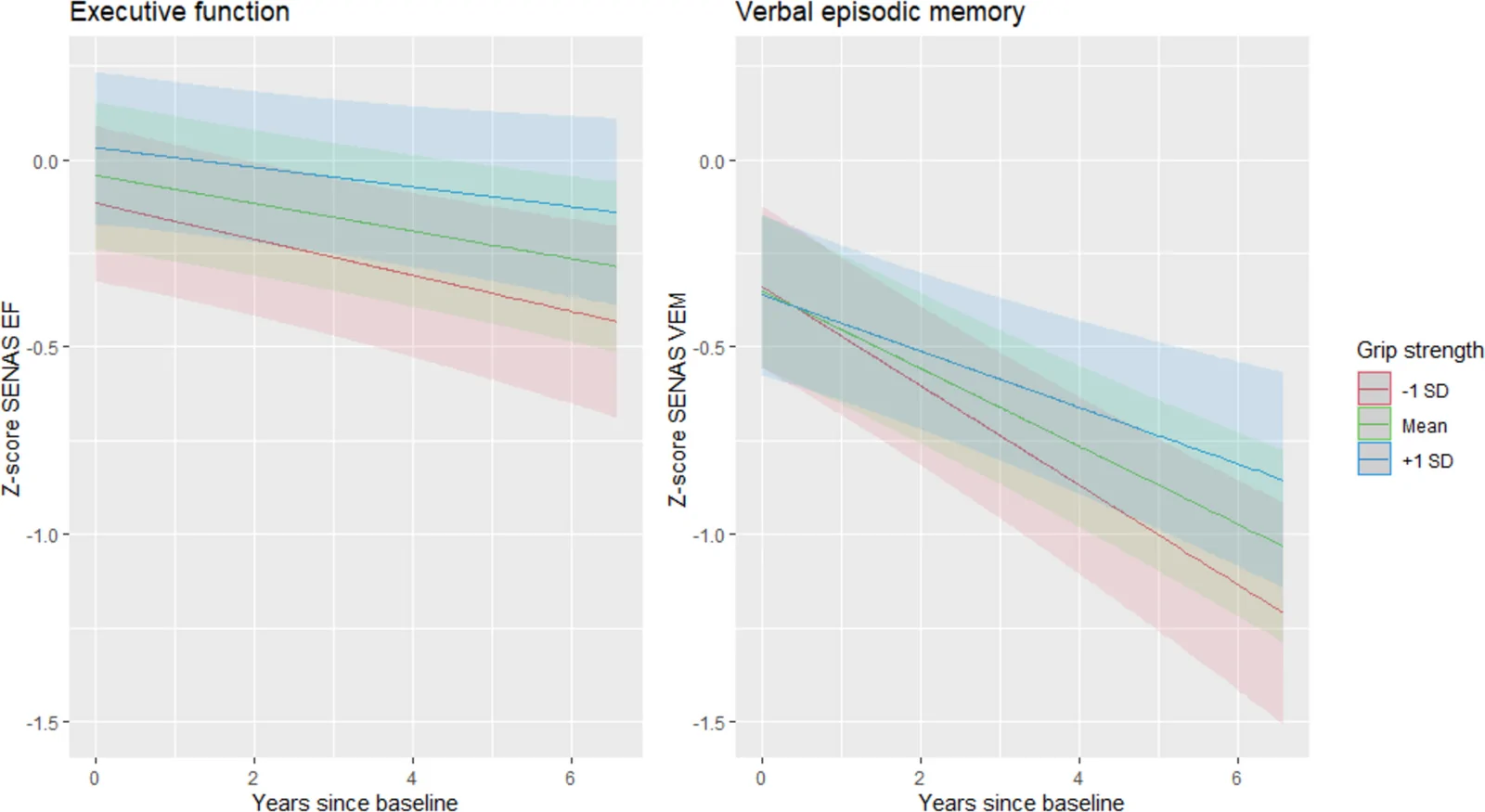
Average trajectories of executive function (EF) and verbal episodic memory (VEM) by grip strength estimated with linear mixed-effects models with random intercepts and inverse probability of censoring weights, and adjusted for age, gender, education, race/ethnicity, time between grip strength and neuroimaging measurement, interview mode, practice effects, and cohort

In multivariable IPCW models, greater grip strength was associated with slightly slower decline in verbal episodic memory after adjusting for age, gender, education, race/ethnicity, time between grip strength and neuroimaging measurement, interview mode, practice effects, and cohort (β = 0.03; 95% CI 0.01, 0.05; Table 2; Fig. 2). However, while significant, the lower confidence limit approached 0. There was no significant association between grip strength and executive function decline (β = 0.01; 95% CI − 0.01, 0.03).

**Table 2:** Association of grip strength with cognitive function over time

Cognitive outcome	β (95% CI)
Executive function	0.01 (−0.01, 0.03)
Verbal episodic memory	0.03 (0.01, 0.05)

Estimates were derived from inverse probability of censoring weighted linear mixed effects models for the grip strength-by-time interaction with random intercepts. Models adjusted for age, gender, education, race/ethnicity, time between grip strength and neuroimaging measurement, interview mode, practice effects, and cohort

### Cross-sectional associations of grip strength with neuroimaging markers at baseline

In cross-sectional analyses, greater grip strength was associated with greater total gray matter volume at baseline (β = 0.09; 95% CI 0.03, 0.16; Table 3) after controlling for age, gender, education, race/ethnicity, time between grip strength and neuroimaging measurement, and cohort. Although not significant, the point estimate of the association between grip strength and hippocampal volume was positive (β = 0.03; 95% CI − 0.03, 0.10), and the point estimate with WMH volume was negative (β =  − 0.02; 95% CI − 0.09, 0.04). Results were similar after excluding 78 participants with more than a 1-year gap between neuroimaging and grip strength measures (Table 2).

**Table 3:** Cross-sectional associations of grip strength with brain biomarkers at baseline

	Full population (*n* = 861)	Excluding *n* = 78 with > ± 1 year difference in neuroimaging and grip strength measurements (*n* = 783)
Brain biomarker outcome	β (95% CI)	β (95% CI)
Total gray matter volume	0.09 (0.03, 0.16)	0.12 (0.05, 0.19)
Total hippocampal volume	0.03 (− 0.03, 0.10)	0.05 (− 0.02, 0.11)
Total WMH volume	− 0.02 (− 0.09, 0.04)	− 0.00 (− 0.07, 0.06)

Estimates were derived from linear regression models, adjusted for age, gender, education, race/ethnicity, time between grip strength and neuroimaging measurement, and cohort

### Moderating role of gender and age

In moderation analyses, we did not find significant interactions of grip strength with gender in relation to cognitive and imaging outcomes (Supplementary Figs. 1 and 2; p’s-interaction ≥ 0.48). However, our estimates for women were of greater magnitude compared to men for both cognitive change (Supplementary Fig. 1) and baseline neuroimaging outcomes (Supplementary Fig. 2). When testing by age groups, there was a significant interaction of age-by-grip strength for executive function change (p-interaction = 0.02) but not in relation to  verbal episodic memory change (p-interaction = 0.28; Supplementary Fig. 3). There was a significant interaction for age-by-grip strength in relation to WMH where the point estimates were negative for those under 80 years old and positive among those aged 80 + (p-interaction = 0.02; Supplementary Fig. 4). There were no significant differences for baseline gray matter or hippocampal volume outcomes (p’s-interaction ≥ 0.16; Supplementary Fig. 4).

### Grip strength as a proxy for cognitive resilience

Grip strength was a significant cognitive resilience proxy for WMH volume in relation to verbal episodic memory (p-interaction = 0.04; Supplementary Table 3). After stratifying by grip strength, those with greater levels of WMH volume and lower levels of grip strength had the steepest average declines in verbal episodic memory (1 SD below *z*-scored grip strength: β =  − 0.04, 95% CI − 0.06, − 0.01; Supplementary Fig. 5). Grip strength was not a significant proxy for cognitive resilience for other combinations; we did not find significant interactions of grip strength with total gray matter volume or hippocampal relation to either cognitive outcome, and WMH in relation to executive function (p's-interaction ≥ 0.08; Supplementary Table 3; Supplementary Figs. 5 and 6).

## Discussion

In this study of 861 participants, greater grip strength was associated with slower decline in verbal episodic memory, and cross-sectionally associated with greater total gray matter volume. While results were not significant, the point estimates of grip strength were positive for executive function decline, positive for hippocampal volume, and negative for WMH volume. In moderation analyses, estimates were stronger for women compared with men, and among those under 80 years of age. Lastly, grip strength was a significant proxy for cognitive resilience in relation to WMH and verbal episodic memory, but not other forms of cognitive resilience.

Our findings align with prior literature which found associations of grip strength with total brain volume and change in cognition [6, 44–48]. For example, in the Framingham Offspring Study including 2176 participants, weaker grip strength was associated with lower total brain volume [48]. In a UK Biobank study of 190,406 participants, reduced hand grip strength was associated with greater risk of dementia, poorer cognitive performance, and greater WMH volume [6]. They also found differences among women compared with men for both cognitive function and neuroimaging outcomes [6], which aligns with our findings.

We did not find significant associations of grip strength with hippocampal and WMH volumes, contrary to prior literature [10, 14]. In a separate UK Biobank study of over 40,000 participants, greater grip strength was associated with greater cognitive function and increased regional gray matter volumes, particularly in the thalamus, striatum, and hippocampus [10]. In a separate harmonized analysis including 338 participants (mean age = 93 years) from the 90 + Study and the European Medical Information for Alzheimer’s disease (EMIF-AF) 90 + Study in the Netherlands, greater baseline grip strength was associated with slower cognitive decline and less evidence of neurodegeneration measured by hippocampal, WM lesion, and gray matter volumes [14]. This differs from our effect modification findings where greater grip strength had an expected negative estimate on WMH for those aged < 80 years but a positive estimate for those aged 80 +. While our observed estimates were in the expected direction, differences with prior work may be explained, in part, by selective survival, our modest sample size of 861 participants, and age of participants in our study (median = 77.3 years; range = 53.5, 103). Despite these differences, we expand upon the cognitive resilience literature by using a diverse population spanning a wide age range.

We found that grip strength was a significant proxy for cognitive resilience for WMH and verbal episodic memory, shown by significant moderation. Our results should be interpreted with caution given imprecise estimates and study design limitations which we highlight below. Our null findings for other grip strength-by-neuroimaging-by-time interactions may be partially attributed to low power due to our short average follow-up of 1.63 years among 861 participants. Other factors, such as education [49, 50] and cognitive activities [51], have been demonstrated as proxies that contribute to cognitive reserve. As interactions are already commonly underpowered [52], larger studies with extended follow-up are needed to examine other potentially modifiable cognitive reserve proxies. It is also plausible that there is no such association or potential for reverse causation if loss of integrity in motor control-related brain regions, reduces motor control, thus reducing grip strength.

There are several postulated pathways in which grip strength could be a marker for neurodegeneration. Lower grip strength may be associated with reduced physical activity levels and other poor health behaviors which in turn increase the risk of cardiovascular risk factors and disease [22, 23]. These cardiovascular disease–related factors are associated with cognitive decline [15, 18–20], and markers of neurodegeneration [16, 17]. While we were not able to examine the effects or presence of cardiovascular and metabolic conditions such as hypertension here, future work should assess their role in these associations due to known implications in cognitive and brain aging [15]. Moreover, grip strength and cognition are possibly affected by common underlying neurodegenerative processes [29].

This study has several limitations. First, we cannot infer causality given the limitations of our study design with a short average follow-up of 1.6 years and differences in measurement timing between grip strength and imaging. Given the short follow-up, we were unable to examine associations of grip strength with long-term cognitive change. Our grip strength and neuroimaging measures were assessed at 1 timepoint, while cognitive function was measured repeatedly. Repeated assessment across all data would allow for more thorough testing of alternative pathways [14], including if associations between grip strength and cognition are mediated through neuroimaging, or if the degree of cognitive impairment affects subsequent trajectories of physical function [47], which we were unable to test here. We attempted to account for the differences in the timing of measurement by adjusting for the time between grip strength and neuroimaging in our models. We also included a sensitivity analysis excluding 78 participants with more than a 1-year gap between neuroimaging and grip strength measures. This warrants future work with contemporaneous measures and extended follow-up to detect cognitive decline with accurate temporal order. Lastly, there may be limited generalizability of our findings given that participants all had access to healthcare as long-term members of KPNC [53].

Our study had multiple strengths. We used a unique study population, harmonized across three separate cohort studies of racially and ethnically diverse adults spanning a wide age range who had data on neuroimaging and repeated measures of cognitive function. Findings accounted for selection bias due to loss to follow-up using inverse probability weighting in cognitive change models. Lastly, our outcome measures of cognitive function were psychometrically validated in diverse populations [41, 42].

In this prospective pooled cohort study of 861 older adults, greater grip strength was associated with slower declines in cognitive function and less neurodegeneration. These results suggest that physical function is potentially implicated in cognitive and brain health in early older age and beyond. Importantly, our results are not causal and should be interpreted with caution due to study design limitations. Nonetheless, given the growing older adult population, it is critical to quantify how indicators of physical function relate to cognitive and brain health as they could identify a prevention strategy to delay the onset of cognitive impairment. Further work on grip strength and other markers of physical function is needed to inform physical performance-related interventions and policy to reduce the burden of cognitive decline.

## Supplementary Information

Below is the link to the electronic supplementary material.

ESM 1 (DOCX 130 KB)

## Data Availability

Data are available upon approved request at https://sites.google.com/g.ucla.edu/khandle-study-site/home.
