# Supplementary material for: Is grip strength a marker for cognitive function, neurodegeneration, and resilience?
Source: GeroScience. 2025 Dec 23;48(5):7377–88. doi: 10.1007/s11357-025-02057-y (PMC13601401; doi:10.1007/s11357-025-02057-y)
Supplement: Supplementary file 1 [file 11357_2025_2057_MOESM1_ESM.docx]

# **SUPPLEMENTARY FILE**

**Title:** Is grip strength a marker for cognitive function, neurodegeneration, and resilience?

**Authors and affiliations:** Alexander Ivan B. Posis^1*^, Hilary L. Colbeth^1^, Mihika S. Deshpande^1^, Kristen M. George^1^, Batool Rizvi^1^, Rifat B. Alam^1^, Ruijia Chen^2^, Dan M. Mungas^3^, Paola Gilsanz^4^, María M. Corrada^5,6^, Rachel A. Whitmer^1^

^1^ Department of Public Health Sciences, University of California, Davis, Davis, CA, USA

^2^ Department of Epidemiology, Boston University School of Public Health, Boston, MA, USA

^3^ Department of Neurology, University of California, Davis, Davis, CA, USA

^4^ Kaiser Permanente Northern California Division of Research, Pleasanton, CA, USA

^5^ Department of Neurology, University of California, Irvine, Irvine, CA, USA

^6^ Department of Epidemiology and Biostatistics, University of California, Irvine, Irvine, CA, USA

***Corresponding author:** Alexander Ivan B. Posis, PhD, Department of Public Health Sciences, Medical Sciences 1-C, One Shield’s Ave, Davis, CA 95616, USA (abposis@health.ucdavis.edu).

| Supplementary Table 1: Mean and standard deviation (SD) of average grip strength across three measures by cohort and gender at baseline | | | |
| --- | --- | --- | --- |
| Gender | Cohort | n | mean (SD) |
| Women | STAR | 155 | 23.08 (6.93) |
|  | KHANDLE | 238 | 19.37 (6.28) |
|  | LA90 | 126 | 13.29 (4.50) |
| Men | STAR | 71 | 32.6 (11.51) |
|  | KHANDLE | 186 | 31.03 (8.26) |
|  | LA90 | 92 | 23.35 (7.48) |
| Abbreviations: KHANDLE = Kaiser Healthy Aging and Diverse Lifecourse Experiences; STAR = Study of Healthy Aging in African Americans; LA90 = LifeAfter90 | | | |

| Supplementary Table 2: Baseline participant characteristics by timing of neuroimaging and grip strength measurements | | | | |
| --- | --- | --- | --- | --- |
| Characteristic |  | >±1 year difference in neuroimaging and grip strength measurements (N=78) | Included (N=783) | Overall (N=861) |
| Age | Median [min., max.] | 78.4 [60.7, 101] | 77.3 [53.5, 103] | 77.3 [53.5, 103] |
| Gender | Men | 40 (51.3%) | 306 (39.1%) | 346 (40.2%) |
|  | Women | 38 (48.7%) | 477 (60.9%) | 515 (59.8%) |
| Race/ethnicity | African American/Black | 36 (46.2%) | 324 (41.4%) | 360 (41.8%) |
|  | Asian | 10 (12.8%) | 149 (19.0%) | 159 (18.5%) |
|  | Latine | 16 (20.5%) | 126 (16.1%) | 142 (16.5%) |
|  | Multiracial/ Other | 0 (0%) | 15 (1.9%) | 15 (1.7%) |
|  | White | 16 (20.5%) | 169 (21.6%) | 185 (21.5%) |
| Education | ≤High School/GED | 9 (11.5%) | 112 (14.3%) | 121 (14.1%) |
|  | Some College or Tech/Trade School | 34 (43.6%) | 285 (36.4%) | 319 (37.1%) |
|  | College | 15 (19.2%) | 220 (28.1%) | 235 (27.3%) |
|  | Graduate School | 20 (25.6%) | 166 (21.2%) | 186 (21.6%) |
| Grip strength^a^ | Median [min., max.] | -0.185 [-2.37, 2.06] | -0.0596 [-2.77, 3.39] | -0.0644 [-2.77, 3.39] |
| Note: ^a^Grip strength is z-scored by gender and cohort, thus resulting in positive and negative values | | | |  |

| Supplementary Table 3: Tests of interaction for brain biomarkers-by-grip strength-by-time with cognitive function over time for assessing whether grip strength was a cognitive resilience proxy | | | |
| --- | --- | --- | --- |
| Cognitive outcome | Brain biomarker | χ2 statistic | p-value |
| Executive function | Total gray matter volume | 2.06 | 0.56 |
|  | Total hippocampal volume | 3.47 | 0.33 |
|  | Total WMH volume | 2.05 | 0.56 |
| Verbal episodic memory | Total gray matter volume | 5.26 | 0.15 |
|  | Total hippocampal volume | 6.87 | 0.08 |
|  | Total WMH volume | 8.22 | 0.04 |
| Note: P-values for interaction were derived from likelihood ratio tests comparing linear mixed effects models with and without the brain biomarker-by-time interaction. Models included random intercepts and inverse probability of censoring weights, and adjusted for age, gender, education, race/ethnicity, time between grip strength and neuroimaging measurement, practice effects, and cohort. | | | |

Supplementary Figure 1: Association of grip strength with cognitive function over time, stratified by gender. Estimates were derived from linear mixed-effects models with random intercepts and inverse probability of censoring weights, and adjusted for age, education, race/ethnicity, time between grip strength and neuroimaging measurement, interview mode, practice effects, and cohort. P-interaction was derived from likelihood ratio tests comparing models with and without a grip strength-by-gender-by-time interaction.

Supplementary Figure 2: Cross-sectional associations of grip strength with brain biomarkers at baseline stratified by gender. Estimates were derived from linear regression models, adjusted for age, education, race/ethnicity, time between grip strength and neuroimaging measurement, and cohort. P-interaction was derived from likelihood ratio tests comparing models with and without a grip strength-by-gender interaction.

Supplementary Figure 3: Association of grip strength with cognitive function over time, stratified by age group. Estimates were derived from linear mixed-effects models with random intercepts and inverse probability of censoring weights, and adjusted for age, gender, education, time between grip strength and neuroimaging measurement, interview mode, practice effects, and cohort. P-interaction was derived from likelihood ratio tests comparing models with and without a grip strength-by-age-by-time interaction.

Supplementary Figure 4: Cross-sectional associations of grip strength with brain biomarkers at baseline stratified by age group. Estimates were derived from linear regression models, adjusted for age, gender, education, time between grip strength and neuroimaging measurement, and cohort. P-interaction was derived from likelihood ratio tests comparing models with and without a grip strength-by-gender interaction.

Supplementary Figure 5: Average trajectories of verbal episodic memory (VEM) by total gray matter (GM), hippocampal or log white matter hyperintensities (WMH) and grip strength estimated with linear mixed-effects models with random intercepts and inverse probability of censoring weights, and adjusted for age, gender, education, race/ethnicity, time between grip strength and neuroimaging measurement, interview mode, practice effects, and cohort.

Supplementary Figure 6: Average trajectories of executive function (EF) by total gray matter (GM), hippocampal or log-white matter hyperintensities (WMH) and grip strength, estimated with linear mixed-effects models with random intercepts and inverse probability of censoring weights, and adjusted for age, gender, education, race/ethnicity, time between grip strength and neuroimaging measurement, interview mode, practice effects, and cohort.
